# Supplementary material for: The Effectiveness of Mobile Cloud 12-Lead Electrocardiogram Transmission System in Patients with ST-Segment Elevation Myocardial Infarction
Source: Medicina (Kaunas). 2022 Feb 6;58(2):247. doi: 10.3390/medicina58020247 (PMC8876768; doi:10.3390/medicina58020247)
Supplement: Supplementary file 1 [file medicina-58-00247-s001.zip › medicina-1567789-supplementary.pdf]

medicina MDPI

Table S1. Univariate analysis to predict more than 15-day hospital stay.

Variables	OR	95% Confidence Interval	*p*-value
Transport distance	1.02	0.93 – 1.07	0.96
Arrival in off hour	1.50	0.46 – 4.87	0.5
Collapse-to-balloon time	1.00	1.00 – 1.01	0.7
Door-to-catheter laboratory time	0.99	0.97 – 1.02	0.5
Door-to-balloon time	1.00	0.98 – 1.02	0.8
FMCTB time	1.00	0.97 – 1.02	0.7
Current or past smoker	1.20	0.37 – 3.96	0.8
Complication of heart failure after PCI	3.18	0.83 – 12.3	0.1

FMCTB: first medical contact-to-balloon, PCI: percutaneous coronary intervention.
